# Host Cell Receptors Implicated in the Cellular Tropism of BVDV

**DOI:** 10.3390/v14102302

**Published:** 2022-10-20

**Authors:** Shuhui Qi, Lijing Wo, Chao Sun, Juan Zhang, Quanhai Pang, Xin Yin

**Affiliations:** 1 State Key Laboratory of Veterinary Biotechnology, Harbin Veterinary Research Institute, Chinese Academy of Agricultural Sciences, Harbin 150069, China; 2 College of Animal Science and Veterinary Medicine, Shanxi Agricultural University, Jinzhong 030801, China

**Keywords:** BVDV, receptor, CD46, HS, LDL receptor, ADAM17, viral tropism

## Abstract

Bovine viral diarrhea virus (BVDV) is one of the most hazardous viruses, which causes huge economic losses in the cattle industry around the world. In recent years, there has been a continuous increase in the diversity of pestivirus worldwide. As a member of the genus *Pestivirus* in the *Flaviviridae* family, BVDV has a wide range of host animals including cattle, goat, sheep, pig, camel and other cloven-hoofed animals, and it has multi-tissue tropism as well. The recognition of their permissive cells by viruses via interaction with the cellular receptors is a prerequisite for successful infection. So far, little is known about the cellular receptors essential for BVDV entry and their detailed functions during BVDV infection. Thus, discovery of the cellular receptors involved in the entry of BVDV and other pestiviruses is significant for development of the novel intervention. The viral envelope glycoprotein E^rns^ and E2 are crucial determinants of the cellular tropism of BVDV. The cellular proteins bound with E^rns^ and E2 potentially participate in BVDV entry, and their abundance might determine the cellular tropism of BVDV. Here, we summarize current knowledge regarding the cellular molecules have been described for BVDV entry, such as, complement regulatory protein 46 (CD46), heparan sulfate (HS), the low-density lipoprotein (LDL) receptor, and a disintegrin and metalloproteinase 17 (ADAM17). Furthermore, we focus on their implications of the recently identified cellular receptors for pestiviruses in BVDV life cycle. This knowledge provides a theoretical basis for BVDV prevention and treatment by targeting the cellular receptors essential for BVDV infection.

## 1. Introduction

Bovine viral diarrhea virus (BVDV), a member of the genus *Pestivirus* of the family *Flaviviridae*, is widespread and causes significant economic losses [1,2]. It has been recognized as the major factor of respiratory and gastrointestinal symptoms in affected cattle. Furthermore, BVDV-susceptible animals also include goat, sheep, camel and pig.

**Figure 1:**
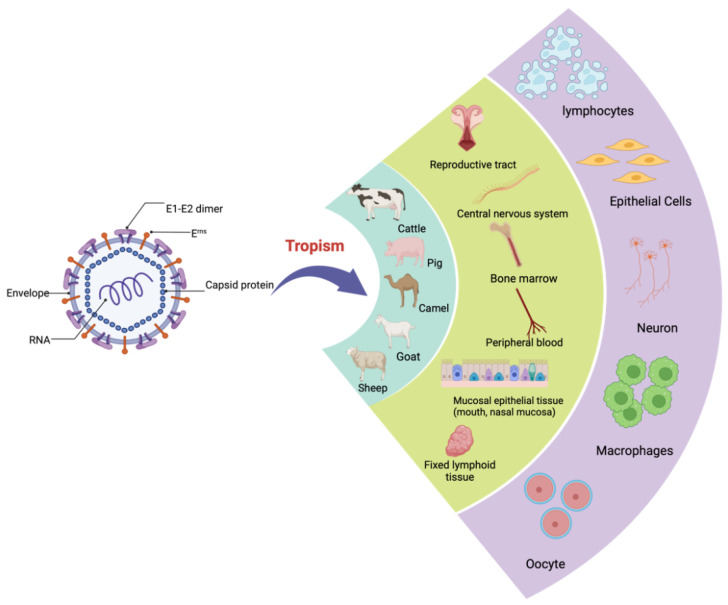
The morphology and tropism of BVDV. An illustration of the BVDV particle shows the morphology and tropism of the virus. Capsid proteins enclose viral RNA. The outer viral envelope protein contains E^rns^ and E1-E2 heterodimers, which are required for the virus entry. BVDV can infect a variety of animals (such as cattle, pig, camel, goat, and sheep) with a wide range of multi-tissue tropism (reproductive tract, central nervous system, bone marrow, peripheral blood, mucosal epithelial tissue and lymphoid tissue). Lymphocytes, epithelial cells, neurons, oocytes and macrophages are common susceptible cells.

The particles of BVDV are spherical or semi-spherical in shape. The virion consists of an outer bi-lipid layer envelope surrounding an electron-dense core as revealed by cryo-electron microscopy and negative staining electron microscopy [3,4]. There is some variation in the size of viral particles with a diameter of approximately 50 nm (range between 40 nm and 60 nm) for the majority, but about 2% of the particles show a diameter of ∼65 nm [3,4] (Figure 1).

**Figure 2:**
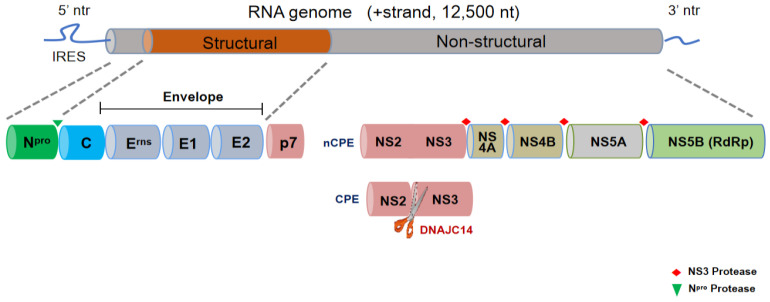
Graphical representation of the genome organization of BVDV. The BVDV genome is a single-stranded RNA of approximately 12.5 kilobases (kb) in size. The central panel shows the composition of the viral genome (the structural and non-structural proteins): four structural proteins (C, E^rns^, E1 and E2) and eight non-structural proteins (N^pro^, p7, NS2, NS3, NS4A, NS4B, NS5A and NS5B). The non-structural protein N^pro^ encodes RNase; NS2 encodes a cysteine-auto-protease that cleaves NS2 and NS3, by which CP and NCP BVDV can be distinguished; NS3 encodes serine protease, RNA helicase and NTPase; NS5A encodes a protein that is a component of viral replicase; and NS5B is encodes RNA-dependent RNA polymerase.

BVDV contains a positive-sense single-stranded RNA genome that is approximately 12.5 kb in length. The genomic RNA of BVDV consists of one long open reading frame (ORF) which encodes a polyprotein of about 4000 amino acids that is co- and post-translationally processed by both viral and cellular proteases into at least 11 mature viral proteins: N^pro^, C, E^rns^, E1, E2, p7, NS2-3, NS4A, NS4B, NS5A and NS5B [5] (Figure 2). C, E^rns^, E1 and E2 belong to the structural proteins which mainly participate in viral particle assembly, while N^pro^, p7, NS2, NS3, NS4A, NS4B, NS5A, NS5B belong to nonstructural proteins that are essential for viral replication. Among those structural proteins, E^rns^, E1 and E2 can be classified as envelope proteins, while C as the capsid protein (capsid protein C). These three envelope proteins are produced as precursor proteins that is sequentially processed to free envelope proteins E^rns^, E1, and E2 through two-step cleavage reactions. Capsid protein C is the most abundant protein in the infected cells, but on the surface of the virion, E2 is the most abundant surface protein and mainly exists in the form of E1-E2 heterodimers [3]. This heterodimers is the most important structure for E2 to excise its function of fusion with host cell membranes [6]. In addition, E2 proteins can form E2-E2 homodimers via intermolecular disulfide bonds formed between their cysteine residues closest to the C-terminus [7]. For nonstructural proteins, cleavage for the release of NS3 at the NS2-3 junction distinguishes cytopathic (CP) and noncytopathic (NCP) biotypes and is achieved by the insertion of viral and host protease target sequences upstream of NS3. Therefore, the generation of NS3 is a hallmark of CP virus strains and their cytopathicity [8,9,10,11,12].

BVDV commonly has tissue tropism [13,14,15]. It has been found to replicate in mucosal epithelial tissue (mouth, nasal mucosa), the central nervous system [13], peripheral blood, bone marrow and fixed lymphoid tissue [14]. In addition, BVDV can infect all major somatic cells in the reproductive tract, such as oocytes [15]. However, the underlying mechanism by which BVDV is able to infect a wide range of tissue needs to be further investigated. Moreover, BVDV is divided into two biotypes, cytopathic (CP) and non-cytopathic (NCP), based on its ability to lyse cells in tissue culture and its role in the lethal mucosal disease (MD) of persistently infected (PI) animals [16]. The syndrome caused by the two biotypes differs mainly in the onset and severity of the disease. Both biotypes are capable of causing disease in cattle. Interestingly, the non-cytopathic strain has a tropism for white blood cells, lymphoid organs and the respiratory tract, while the cytopathic strain is more restricted to the digestive tract [17]. The NCP strain can cause PI in animals, which is widespread in nature [17]. Cytopathic biotypes have only been isolated in outbreaks of mucosal disease (MD), which is caused by mutation of NCP to CP biotypes, usually due to changes in the viral genome (i.e., the recombination between the NS2-NS3-NS4 regions of the viral genome and the host genome). It has been shown that the E2 glycoprotein sequence of BVDV is highly variable [16]. Considering the interaction of BVDV with the host, the insertion of the amino acid in the E2 protein may be the result of the different cellular tropisms required by BVDV in order to expand its tissue tropism. This also implies that variation of the virus at the amino acid level is an important basis to support the idea that the virus remains inherited in different tissues [16].

The interactions between a virion and the cellular receptors on its permissive cells are an important event for virus infection, especially in the beginning of the virus life cycle. The adsorption of the vast majority of viral particles on the cellular surface can be divided into two stages: non-specific adsorption and specific adsorption. Non-specific adsorption of the virus particles mainly relies on electrostatic action to bind to the host cell in contact with the host cell; whereas, the specific adsorption is specifically mediated by the recognition and binding to the corresponding receptor on the host cell membrane. The first step of the specific adsorption is attachment of the virions through its virus attachment proteins (VAP) to the cellular receptor on the permissive cells, and this interaction is thought to often determine the host range and cell tropism of the virus [18]. Usually, there is more than one specific receptor involved in viral entry, and a variety of substances can act as viral receptors, for example, carbohydrates, lipids and proteins. Most viruses attach to cell surface proteins that belong to an immunoglobulin superfamily [18]. However, less is known about cellular mechanisms leading to the entry of BVDV and other related pestiviruses. Until today, only four cellular receptor molecules have been described for BVDV, namely, complement regulatory protein 46 (CD46), heparan sulfate (HS), and the low-density lipoprotein (LDL) receptor. Classical swine fever virus (CSFV) and BVDV belong to the same genus of pestiviruses and ADAM17 is considered as a key receptor for CSFV entry, and it was later found that ADAM17 also had a large impact on BVDV invading cells, however, the most important key receptors for BVDV may remain unrecovered, rendering BVDV receptor exploration particularly important in studying the life cycle of BVDV.

In this review, we summarize our current understanding of the interactions between BVDV and host cells and outline the role and importance of viral and host proteins in viral infection.

## 2. Viral Proteins That Mediate BVDV Entry: E^rns^, E1 and E2

The entry of viruses into cells is the first step in the virus life cycle. The interactions between the glycoprotein on the virions with receptors on permissive cells are thought to determine the host range and cell tropism [19]. Current research has revealed that enveloped viruses have developed various adroit mechanisms to invade their host cells. This process requires one or more viral envelope glycoproteins to achieve cell attachment and membrane fusion [20]. Enveloped viruses usually use two general mechanisms of entry, receptor-mediated endocytosis and fusion at the cell surface [21].

BVDV virions contain three enveloped proteins on the surface, E^rns^, E1 and E2. Previous studies have shown the envelope glycoprotein E2 to be essential for virus infectivity [22]. Furthermore, the formation of BVDV E1-E2 heterodimers is essential, while E^rns^ might be dispensable for virus entry [21]. The ability of chloroquine, bafilomycin A1 or ammonium chloride to inhibit BVDV infection in MDCK cells suggests that BVDV mainly uses clathrin-dependent endocytosis to infect bovine cell lines and the entry is dependent on an acidic environment [23]. More importantly, entry of both BVDV-1 and BVDV-2 into ovine cells also occurred through clathrin-dependent endocytosis, endosomal acidification, and low pH-dependent fusion following an activation step, suggesting the involvement of common cellular receptors for BVDV-1 and BVDV-2 [24].

### 2.1. E^rns^

E^rns^ is a heavily glycosylated except for its C-terminal region that plays multiple roles in BVDV infection, as the amphiphilic helix at C-terminal region acts as a membrane anchor, a signal peptidase cleavage site, and a retention/secretion signal [25]. E^rns^ forms a disulfide-linked dimer of 90 kDa very early upon virus replication. It possesses endoribonuclease activity to cleave viral RNAs, thus evading the activation of IFN responses [26,27]. E^rns^ was also found to be attached to the virion and to membranes within infected cells via its C-terminus, although it lacks a hydrophobic anchor sequence [26,28]. In addition, the integrity of the membrane anchor was found to be important for recovery of infectious virus [26]. Analyses with different extraction procedures showed that E^rns^ is neither easily stripped from the membrane, like a peripheral membrane protein, nor as tightly membrane bound as a trans-membrane protein. Although binding of E^rns^ to glycosaminoglycans and immobilized heparin has been shown, the possibility that a cell- or species-specific receptor existed could not be excluded.

Current studies have explored certain roles of E^rns^ in the entry of BVDV into permissive cells by generating pseudotyped viral particles harboring different combinations of BVDV enveloped proteins. Pseudoviruses that contain E1 and E2 but not E^rns^ were infectious, indicating that E^rns^ is dispensable for virus entry [21]. In contrast, viruses isolated from the MDBK CD46 knockout cell line were able to grew efficiently despite the lack of the entry receptor, the exchange of a cysteine at position 441 in the E^rns^ resulted in a loss of E^rns^ dimerization and likely enhanced viral cell-to-cell spread. It is speculated that the aa exchange at position 479 may allow the virus isolates NADL (BVDV-1a) D02/11-2 (BVDV-1d) and CS8644 (BVDV-2a) to compensate for the loss of the potential binding side CD46 by an increased binding of HS [29]. Therefore, the role of E^rns^ in the process of virus entry remains unclear, and it needs to be further investigated.

### 2.2. E1

E1 is not only the least characterized component of the virion, but also neither its structure, nor its function has been analyzed in detail until now. Glycosylated E1 proteins are about 27–33 kDa depending on the type of BVDV, which is only about half the size of the E2 protein. E1 forms disulfide linked heterodimers with E2 in all pestiviruses, and this structure was suggested to be important as the absence of heterodimers prevent the infectivity of BVDV [21]. However, the exact role of E1 in entering cells is still unknown.

Same as BVDV, hepatitis C virus (HCV), which is a member of *Flaviviridae* family, is made up of a heterodimer consisting of the envelope glycoproteins E1 and E2, which is crucial to the process of virus entry. Interestingly, recent studies suggest that E1 appears to have more than an assisting role for E2 in the process of viral attachment and binding. By analyzing the HCV E1 sequence, one of the protein conserved regions is divided into an N-terminal domain (NTD, 192–239), a putative fusion peptide (pFP, 272–285), a conserved region (CR, 302–329), and a C-terminal transmembrane domain (TMD, 350–381) [30]. The highly conserved pFP is thought to be involved in the fusion of the viral envelope and the plasma membrane upon HCV entry [31,32]. During the entry of HCV into cells, E2 is generally regarded as the primary envelope protein that interacts with host cell receptors. However, mutation in E1 can alter the binding with HCV entry factors from CLDN1 to CLDN6 according to two independent studies [33,34], highlighting the significance of E1 interaction with CLDN1 during HCV infection. It is currently believed that the E1 of both BVDV and hepatitis C virus is a fusion protein. Although conserved hydrophobic sequence in E1 (CSALYVGDLC, residues 272–281) is essential for HCV fusion [35,36,37,38,39,40], the similar hydrophobic sequence in BVDV E1 has not been determined yet. Thus, the role of E1 in the entry of BVDV into cells is unclear and requires further study.

### 2.3. E2

E2 can form homodimers as well as heterodimers with E1 [41]. These two dimers play an essential role in BVDV entering cells and interacting with receptors. E2 protein can form β-hairpin motif structure when binding to the cellular receptors through E2-E2 dimer, it has been verified that the β-hairpin motif is critical for the interaction with host cell receptors by mutating polar residues Asn 144 and Thr 147 to Ala of BVDV E2 [42]. Blocking is effective with either CSFV or BVDV E2 on porcine and bovine cells, suggesting that both CSFV and BVDV viruses potentially share the identical receptor, and E2 proteins play key roles in the process [43]. With regard to CSFV, E2 protein has been extensively studied and defines four antigenic domains located in the N-terminal half in the order of B/C/D/A [44,45]. According to related studies, the E2 protein structure of BVDV is divided into three or four domains, of which I or DA corresponds to the B/C domain of the E2 protein of CSFV, and II or DB corresponds to the D/A domain [20,46]. The A domain of CSFV E2 protein has neutralizing activity, indicating that the II domain of BVDV E2 may have the ability to bind to receptors [47]. Another study demonstrated that the β-hairpin motif exposed in the BVDV E2 domain II mediates receptor binding [42]. Thus, whether there is an epitope similar to the D/A domain in CSFV in BVDV E2 still needs further research.

Previous studies have shown that E2 protein can specifically bind to CD46 receptors on permissive cells [48]. However, in CD46 knockout MDBK cell line, BVDV can still infect MDBK cells, indicate that E2 may bind to other uncharacterized receptors [29,43]. Briefly, E^rns^ and E2 are involved in BVDV infection, which bind with cellular receptors during virus entry and the function of E1 is not clear yet.

## 3. Cellular Proteins That Mediate BVDV Entry

**Figure 3:**
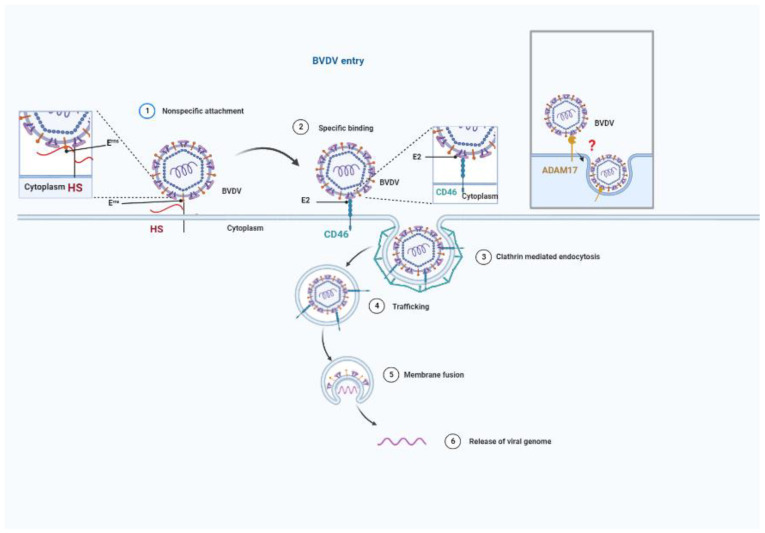
The entry route of BVDV. The entry of BVDV initiates the binding to some cellular membrane proteins, such as HS, CD46 and ADAM17. Of these, HS is thought to be involved in viral non-specific attachment and CD46 is considered to be a crucial receptor for BVDV entry. After viral attachment and binding, BVDV is trafficked by endosomes through clathrin-mediated endocytosis. At low pH, endosomes are acidified to trigger membrane fusion, which unravels the envelope and releases the viral genome into the cytoplasm.

Discovery of the cellular proteins that mediate virus attachment and entry is critical for understanding the virus entry process. Unfortunately, the pestiviral entry process is still poorly understood, despite the previous studies showing BVDV infects host cells through binding to several membrane proteins following by clathrin-dependent endocytosis [23,49]. So far, only four cellular receptor molecules have been described for BVDV, namely, complement regulatory protein 46 (CD46), heparan sulfate (HS), and the low-density lipoprotein (LDL) receptor, and a disintegrin and metalloproteinase 17 (ADAM17) (Figure 3).

### 3.1. Complement Regulatory Protein 46 (CD46)

Complement regulatory protein 46 (CD46) belongs to the family of complement activation regulators whose amino-terminal region consists of variable numbers of tandemly linked cysteine-rich modules of approximately 60 amino acids, termed complement control protein (CCP) repeats [50]. CD46 includes four complement control proteins (CCP1-CCP4) [48,51], a highly variable Serine/Threonine/Proline-rich region (STP), a transmembrane domain (TM) and a cytoplasmic tail (CT).

CD46 is a ubiquitously expressed “multitasker” as it is a regulator of both the complement system and adaptive immunity [52]. For BVDV, bovine complement regulatory protein 46 (CD46_bov_) has been identified as a receptor by blocking infections using anti-CD46_bov_ mAbs or serum, and by CD46-transfection of non-permissive porcine cells [48,53]. Viral entry of BVDV is mediated by interaction of E2 with a minimal essential binding platform that is constituted by two short peptides (E66QIV69 and G82QVLAL87) on antiparallel beta strands within CCP1 [53]. Exchanges of these two peptide sequences were sufficient for a loss of function in CD46_bov_ as well as a gain of function in porcine CD46 (CD46_pig_). Moreover, a study has shown that CD46_bov_ variants with long CTs shift cell permissivity to infection with BVDV-1 strain NADL [54]. A similar dependence on CD46_bov_ was also shown for isolate HaVi-20, a member of the species *Pestivirus H* [55]. Based on antibody-mediated blocking assay, it was suggested that CSFV (*Pestivirus C*) uses CD46_pig_ as a major cellular entry factor [56]. Interestingly, overexpression of CD46 in porcine cells increased susceptibility to BVDV by 100-fold. However, overexpression of CD46 in non-susceptible human or murine cells did not confer susceptibility to BVDV infection, although BVDV RNA replication is supported by these cells. These findings also suggest that a so-far-unknown co-receptor(s) is required for BVDV infection [48].

Previously, it was already noted that the ubiquitous expression of CD46_bov_ is not consistent with the tissue tropism of BVDV [48]. Liebler-Tenorio suggested that BVDV first infects the tonsils and then spreads to other lymphatic tissues. In the late stage of infection, BVDV can be detected in all organs and tissues [57]. This further supports the notion that in addition to CD46, other unidentified molecules play key roles in BVDV entry. Interestingly, CD46bov is present in all nucleated cells and the ability of BVDV-1 to infect polarized airway epithelial cells from the basolateral side where no CD46_bov_ is expressed also points to the existence of an alternative entry factor [58] (Table 1). In addition, in CD46 knockout cells, BVDV can still infect susceptible cells in small amounts. In conclusion, CD46 is not the only receptor that mediates BVDV entry into cells, and it may be co-functioning with other undetermined receptors for BVDV entry.

### 3.2. Heparan Sulfate (HS)

Heparan sulfate (HS) is a linear sulfated glycosaminoglycan (GAG) expressed by virtually all animal cells. It is an ancient molecule that is present in Cnidaria (e.g., Hydra) and all metazoans analyzed to date, with the exception of Porifera [59,60,61]. The mammalian GAG heparan sulfate contains a domain structure comprising undersulfated sequences rich in GlcNAc (where Ac is acetyl) residues (called “NA domains”) and highly sulfated sequences rich in GlcNS (where S is sulfo) residues (called “NS domains”) [61,62,63]. These domains structurally vary based on the species and tissues from which an HS is obtained [64], and the NS domain is of particular importance in cellular behavior and disease processes [65].

Passaging of CSFV in cell culture can lead to increased usage of the cellular glycosaminoglycan (GAG) heparan sulfate (HS) as an attachment factor [56,66,67]. In principle, cell culture adaptation mediated by increased HS binding rarely occurs in BVDV. However, especially under selective pressure through lack of CD46_bov_, BVDV-1 and BVDV-2 are also able to adapt to HS [29,68]. Responsible for this adaptation is a mutation at position 479 in the E^rns^ protein that facilitates enhanced binding to HS [29,66,69], making adapted BVDV-1 and -2 strains independent of their original receptor CD46_bov_ [29]. This mutation is of importance, since the same position (aa 476 in *Pestivirus* C (CSFV) correlates to aa 479 in *Pestivirus* A/B) has been shown to be crucial for CSFV interaction with membrane-associated HS [66]. The exchange of an uncharged aa (glycine) to a positively charged aa (arginine) at this position has been described to increase virus replication in vitro of CSFV variants carrying this mutation [66,70]. It has also been suggested that this aa substitution (position 476 in CSFV; position 479 in BVDV-1 and -2) increases the positive charge of the E^rns^ region and that this particular aa is exposed to the surface and involved into direct binding to the negatively charged HS [66]. Reimann and colleagues [71] also associated the increased virus infectivity in vitro with the same aa substitution of glycine to arginine at position 479 in CP7_E2alf, a chimeric pestivirus constructed from a BVDV-1 backbone (strain CP7) and E2 from CSFV (strain Alfort).

HS and other GAG were also shown to bind a cluster of basic amino acids within the C-terminal domain of the glycoprotein E^rns^ of BVDV strain Pe515 [68,72]. HS has been further described to be important for cellular binding of different viruses, e.g., Schmallenberg virus, hepatitis E virus and rabies virus [73,74,75]. Other viruses use HS or heparan sulfate proteoglycans (HSPG) under in vivo conditions to enter the host cell, and for some viruses, the biological relevance of binding to HS or HSPGs is still controversial [76]. Therefore, in the absence of CD46_bov_, HS acts as an attachment receptor that is similar to CSFV in the process of BVDV infecting cells (Table 1).

### 3.3. Low-Density Lipoprotein (LDL) Receptor

Among several proposed cellular receptors for BVDV, the low-density lipoprotein (LDL) receptor is of special interest because it is also considered a receptor for the related hepatitis C virus (HCV) [77,78]. Further evidence came from the promotion of virus binding to LDL receptor-deficient fibroblasts after expression of the recombinant LDL receptor [79]. Endocytosis of the *Flaviviridae* viruses, HCV, GB virus and Cyhepatitis G virus was shown to be mediated by LDL receptors on cultured cells. Studies using LDL receptor-deficient cells or a cytolytic BVDV system indicated that the LDL receptor may be the main but not exclusive means of cell entry of these viruses [80] (Table 1).

An important role of the LDL receptor in BVDV entry was suggested by an inhibitory effect of an anti-LDL receptor antibody on infection of bovine turbinate (BT) cells with BVDV [80]. In addition, a bovine cell line (CRIB cells) which is completely resistant to BVDV infection [81] was shown to lack a functional LDL receptor [80]. However, in connection with further studies on BVDV entry, Thomas Krey reevaluated the putative role of the LDL receptor as a cellular receptor for BVDV [82]. It was clearly demonstrated that neither of the two monoclonal antibodies against the LDL receptor inhibited BVDV infection of two bovine cell lines [82]. Thus, at present, no solid experimental evidence supports an involvement of the LDL receptor in BVDV invasion.

### 3.4. A Disintegrin and Metalloproteinase 17 (ADAM17)

ADAM17, also named tumor necrosis factor-α-converting enzyme (TACE), belongs to the disintegrin and metalloproteinase (ADAM) family of proteins. This protein family consists of Type-I transmembrane proteins, and is primarily responsible for the processing of many transmembrane proteins. Fei Yuan has already demonstrated that CSFV E2 protein could recognize the metalloproteinase domain to exploit ADAM17 for infection of permissive cells [83]. Marianne Zaruba found that there was no expression of ADAM17 protein on the surface of CRIB cells by comparing the expression of CRIB cells and MDBK cells surface proteins, and through genetic analysis of the two cells, no full-length mRNA of ADAM17 was found in CRIB cells [84]. However, by overexpressing the ADAM17 protein in CRIB cells, it was found that the cell regained susceptibility to the virus. That demonstrates that ADAM17 may act as an essential factor for BVDV entry in permissive cells, but more experiments are needed to prove this conclusion (Table 1).

The absence of the CD46 or HS could not completely block the infection of host cells infected by BVDV, indicating that the key receptor molecules that specifically mediate the invasion of BVDV into target cells are still unknown. Additionally, CD46 or HS may as an attachment or uncoating receptor in BVDV infection, cofunctioning with key receptors that are undiscovered. This remains to be further investigated.

**Table 1:** Cellular proteins that mediate BVDV entry.

Protein	Structure	Function	Notes	References
CD46	Sixty amino acids Belongs to the family of regulators of complement activation	Has been extensively characterized as a receptor for extensive virus CD46 serves as a cofactor for plasma serine protease factor I	Binds to viral E2 protein	[53,54,56,58]
HS	Linear sulfated glycosaminoglycan	Serve as attachment factor for BVDV HS has been described to be important for cellular binding of different viruses	Binds to E^rns^	[66,67,68,75,79,80,81,82]
LDL	Single-stranded glycoprotein Belongs to family of low-density lipoprotein receptor	Regulates the homeostasis of cholesterol	It is also considered a receptor for the related hepatitis C virus	[77,78,79,80]
ADAM17	Belongs to the disintegrin and metalloproteinase (ADAM) family of proteins	Responsible for the processing of many transmembrane proteins	May act as essential factor for BVDV entry	[83,84]

## 4. Current Issues and Future Challenges

With current drugs and vaccines being ineffective in treating and preventing BVDV, studying the entry of BVDV into host cells is the current priority for interrupting BVDV tranmissions by producing genetically modified cows with mutations in the key receptors. BVDV, which has multi-tissue tropism, is bound to have a universal receptor in cells, which facilitates BVDV invasion and infection. The CD46 receptor identified so far, as well as other possible influencing factors—HS and ADAM17, do not explain how BVDV infects multiple different cells. Several studies have shown that CD46 and ADAM17 are widespread in tissue, and HS is expressed by virtually all animal cells [58,59,85]; these resultsare inconsistent with the tissue tropism of BVDV. Further research into the cellular receptors for BVDV entry has important implications for our efforts to block its infection and treat BVDV. With the improvement of high-throughput gene sequencing technology and the wide application of gene editing technologies such as CRISPR, genome-based functional screening technology has been widely used in the discovery of key host factors for virus replication, providing a powerful tool for our systematic understanding of key molecular events in the virus life cycle. To explore other cellular receptors of BVDV, the following methods are provided by combining existing technologies: the construction of bovine whole-genome CRISPR sgRNA library, siRNA pool that concludes receptors which have been identified by other viruses, such as HCV, CSFV and Zika virus, and the functional genes overexpression can effectively help us to screen the influencing factors required for the entry of BVDV. However, this process is equally difficult: The host proteins screened may be crucial proteins for cells for which the corresponding knockdown techniques are not available; Genes of differential factors may be mutated or species-specific, which makes screening for entry receptors even more uncomfortably; The inability to validate the screened receptor proteins effectively also plagues further studies, and conventional electron microscopic observation and laser confocal are insufficient for the purposes of such work.

In addition, transcriptome high-throughput analysis of gene expression in different tissues or cells could provide another idea for finding potential receptors. When different tissues are infected with BVDV, the expression of some proteins is increased and the opposite is true for others. However, this approach also has shortcomings. Species specificity, cell specificity and other factors make it difficult to accurately measure the level of entry receptors. Changes in protein expression may be generated by the non-entry pathway of BVDV, which also disturbs the verification of receptors. Briefly, the uncertainty of the receptor for BVDV entry adds to the difficulty of interrupting its infection and will be an important challenge when focusing on the treatment of BVDV by targeting the entry process.

## 5. Conclusion

In conclusion, the entry of BVDV into target cells involves envelope glycoproteins E2 and E^rns^ and through interactions with CD46 and HS receptor, respectively. However, the absence of these receptors could not completely block the infection of host cells infected by BVDV, indicating that the key receptor molecules that specifically mediate the invasion of BVDV into target cells are still unknown. This remains to be further investigated and will help us better understand the viral tropism.
